# Haemodynamic Early Outcomes of Sinus Plication for Bicuspid Aortic Valve Repair

**DOI:** 10.1093/icvts/ivag103

**Published:** 2026-04-10

**Authors:** Kay Maeda, Nao Ichihara, Toshio Baba, Tomonobu Abe, Atsushi Yamaguchi, Dai Une, Mikizou Nakai, Hiroshi Matsuura, Takeshiro Fujii, Takashi Kunihara

**Affiliations:** Department of Cardiac Surgery, The Jikei University School of Medicine, Tokyo, 105-8461, Japan; Department of Cardiac Surgery, The Jikei University School of Medicine, Tokyo, 105-8461, Japan; Department of Cardiovascular Surgery, Bellland General Hospital, Sakai, 599-8247, Japan; Division of Cardiovascular Surgery, Department of General Surgical Science, Gunma University, Maebashi, 371-8511, Japan; Department of Cardiovascular Surgery, Saitama Medical Center, Jichi Medical University, Saitama, 330-8503, Japan; Department of Cardiovascular Surgery, Kawasaki Medical School, Okayama, 701-0192, Japan; Department of Cardiovascular Surgery, National Hospital Organization Okayama Medical Center, Okayama, 701-1192, Japan; Department of Cardiovascular Surgery, NTT Higashi Nihon Sapporo Hospital, Sapporo, 060-0061, Japan; Division of Cardiovascular Surgery, Department of Surgery, Toho University Faculty of Medicine, Tokyo, 143-8541, Japan; Department of Cardiac Surgery, The Jikei University School of Medicine, Tokyo, 105-8461, Japan

**Keywords:** aortic valve repair, bicuspid aortic valve, sinus plication

## Abstract

**Objectives:**

Postoperative stenosis negatively affects durability after bicuspid aortic valve (BAV) valvuloplasty. This study was performed to evaluate the haemodynamic efficacy of sinus plication (SP) to avoid stenosis after asymmetrical BAV repair.

**Methods:**

Between 2018 and 2023, 41 patients with BAV (33.3 ± 11.5 years, 40 males) with moderate-severe aortic regurgitation (AR) underwent aortic valve repair with (SP group, *n* = 14) or without (NSP group, *n* = 27) SP. SP was performed to reduce the circumference of the fused sinus and restore sinus contour symmetry. Preoperative and postoperative haemodynamic parameters were assessed at discharge and 1 year using transthoracic echocardiography.

**Results:**

No deaths occurred within 1 year after surgery in either group. AR was well controlled and adequate left ventricular reverse remodelling was observed in both the groups up to 1 year postoperatively. At 1 year, lower transvalvular peak pressure gradient (PG) and maximum transvalvular flow velocity (Vmax) were observed in the SP group (peak PG: 17.7 ± 5.5 mmHg; % change −8.8% ± 1.1%, Vmax: 1.9 ± 0.31 m/s; % change −13.1% ± 2.1%) compared with the NSP group (peak PG: 24.7 ± 9.6 mmHg; +8.8% ± 1.3%, Vmax: 2.58 ± 0.62 m/s; +9.3% ± 2.5%), while there were no clear differences in % changes of peak PG or Vmax between the groups after adjustment for covariates.

**Conclusions:**

In BAV repair, SP was potentially associated with lower peak PG and Vmax at 1 year. These findings suggest that SP may enhance haemodynamic stability in BAV repair.

## Graphical abstract

**Figure:**
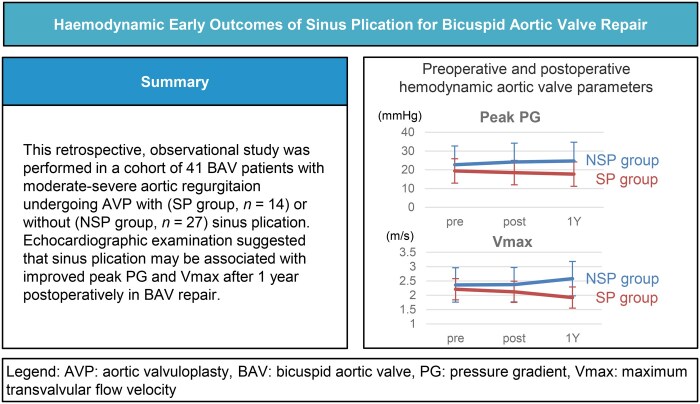

## INTRODUCTION

Bicuspid aortic valve (BAV) is a congenital valve anomaly that can lead to complications, including aortic regurgitation (AR) and aortic stenosis (AS).^1^ Aortic valvuloplasty (AVP) repair is increasingly performed as an alternative to valve replacement to avoid prosthesis-related complications, but achieving reproducible and durable repair remains challenging. BAV repair is attracting attention due to increased understanding of BAV anatomy and advances in surgical techniques. Cusp repair techniques such as plication, triangular resection, and free-margin reinforcement have shown good long-term results.^2^^,^^3^ However, concerns have emerged regarding increased transvalvular pressure gradient (PG) and the need for reoperation after BAV repair. Reproducibility and durability of repair are still surgically challenging, even in experienced centres.^4^^,^^5^

Commissural orientation and root configuration significantly affect repair durability, possibly due to stress distribution in certain phenotypes of bicuspid anatomy.^5–8^ In patients with BAV, asymmetric commissural orientation is associated with poorer haemodynamics and inferior outcomes after AVP because greater cusp plication leads to higher cusp tension. To restore symmetry, double annuloplasty was developed to stabilize the aortic annulus and approach a 180° commissural angle,^9^ although excessive plication in markedly asymmetric valves may lead to postoperative stenosis.^10^^,^^11^ As sinus geometry also influences valve dynamics,^8^ selective correction of the fused sinus has been proposed. Sinus plication (SP), a promising repair technique for asymmetric BAV to address unfavourable commissural orientation and root configuration,^12^ is expected to relieve cusp tension by reducing root circumference in the fused cusp and modifying commissural orientation. Previously, we confirmed haemodynamic efficacy of SP in a pulsatile flow simulation model.^13^ SP in the BAV model significantly decreased AR fraction and improved valve motion with modified commissural angle and reduced transvalvular PG.^13^ Based on these findings, we clinically applied SP in asymmetric BAV repair. Here, we assessed the haemodynamic early impact of SP and the possibility of improved valve durability in patients undergoing BAV repair.

## METHODS

### Ethical statement

This multi-institutional collaborative study was centrally reviewed and approved by the Ethics Committee of The Jikei University School of Medicine (approval No. #35-383[12020]). After approval on March 11, 2024, the directors of each participating institution granted permission for use of their data. Written informed consent was obtained from recent patients, while the requirement for informed consent was waived for earlier patients, for whom opt-out information was provided.

### Patients and study design

This retrospective, observational, multicentre study was performed in a cohort of 41 patients with BAV with moderate-severe AR undergoing elective AVP by a single surgeon (T.K.) between January 2018 and December 2023 at The Jikei University Hospital and 7 other associated institutions. Patients with AS, history of previous cardiac surgery, or aortic root replacement were excluded.

Surgical intervention for AR was indicated according to the European Society of Cardiology guidelines for BAV.^14^^,^^15^ AR severity was quantified by echocardiography using standard parameters (vena contracta width, regurgitant volume, regurgitant fraction, and effective regurgitant orifice area). Moderate AR alone was not considered a surgical indication. Patients with moderate AR underwent surgery only in the presence of additional indications, such as aortic root aneurysm, progressive LV dilation, or planned concomitant cardiac surgery, and symptoms attributable to AR.

All patients underwent transthoracic echocardiography before surgery, at discharge, and 1 year after surgery. Transthoracic echocardiography data included root dimensions, aortic valve area (AVA), left ventricular end-diastolic diameter (LVEDd), left ventricular end-systolic diameter (LVEDs), left ventricular end-diastolic volume (LVEDv), left ventricular end-systolic volume (LVESv), left ventricular mass index (LVMI), transvalvular peak/mean PG, and maximum transvalvular flow velocity (Vmax). AR was determined using colour Doppler echocardiography.^16^

Intraoperative transoesophageal echocardiography (TEE) was performed in all patients to evaluate root configuration, including ventriculoaortic junction (VAJ), sinus of Valsalva, and sinotubular junction (STJ). Valve anatomy was examined with regard to commissural orientation and commissure angle.

The collection, storage, and secondary use of clinical data were conducted in accordance with the World Medical Association Declaration of Taipei. The establishment and ongoing use of the study database were approved and monitored by the Ethics Committee of The Jikei University School of Medicine.

### Surgical techniques

All operations were performed through median sternotomy. After establishment of cardiopulmonary bypass and aortic cross-clamping, the proximal ascending aorta was incised. Cold blood cardioplegia was administered directly into each coronary ostium. The incision was then extended transversely just above the STJ to provide optimal exposure. Annulus dimensions were assessed by direct intubation with a Hegar dilator (TK-sizer; MA Corporation, Japan, distributed by JP Creed Corporation, Japan).^17^ Geometric height (gH) and free margin length of each cusp were measured for quantitative assessment of cusp configuration.^18^ Annular stabilization was performed by external suture annuloplasty using a CV-0 suture (W.L. Gore & Associates, Newark, DE, United States) with a Hegar dilator selected to match target effective annular diameter,^19^ corresponding to the measured geometric height to create sufficient coaptation length.^20^ Central plicating sutures or triangle resection was performed on the cusp free margin until both cusps became identical in height. The effective height (eH) of both cusps was measured and corrected by additional plication of the free margin in cases with eH < 9 mm. In cases with extensive pathology, we performed cusp slicing, triangular resection, or application of an autologous pericardial patch. If cusp bulging was suspected, additional plicating sutures were added on the central portion of the cusp leaflets. Cusp configuration was assessed visually using a surgical microscope (Karl Storz, Tuttlingen, Germany) through the vascular prosthesis in which cardioplegic fluid was injected at a pressure of 50-60 mmHg, and additional manoeuvers were performed if necessary. A second cross-clamping and repair was performed immediately in cases where TEE after aortic unclamping showed eccentric AR.

### Sinus plication

**Figure 1.**
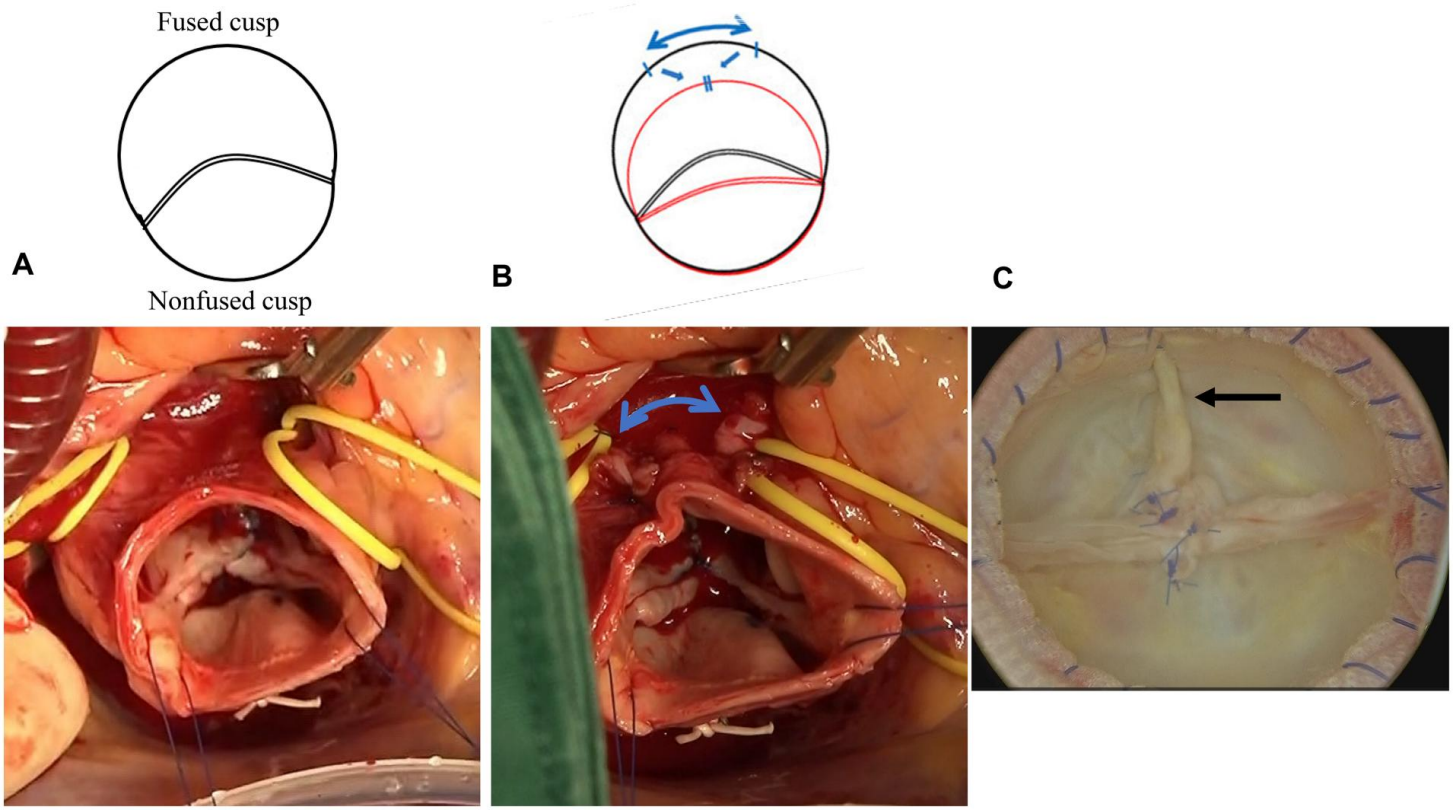
Sinus Plication. (A) Intraoperative view of asymmetric bicuspid aortic valve after cusp repair. (B) Sinus plication of the dilated walls of the fused sinus of Valsalva using a pericardial patch. (C) Surgical microscopic view from inside the sinus of Valsalva after sinus plication (arrow)

After annuloplasty and cusp repair, SP was added for asymmetrically dilated walls of the fused sinus of Valsalva in the SP group.^12^ In our cohort, SP was performed when asymmetric dilatation of the fused sinus was identified on preoperative imaging or intraoperative inspection. Asymmetry was defined as fused sinus diameter ≥1.2 times the non-fused sinus, commissural angle <160°, or marked root contour eccentricity. Intraoperatively, SP was considered when the fused sinus appeared bulging or distorted, resulting in unequal intercommissural spacing or leaflet malcoaptation. This technique was introduced from 2021 and applied thereafter to improve root symmetry. The plication was made along the midpoint of the fused sinus, extending from the annular level towards the STJ. Two mattress sutures using fresh autologous pericardial strips were placed on the outside sinus walls, reducing the basal sinus circumference by approximately 1.5 cm (**Figure 1, Supplementary Video**). The 1.5-cm width represented the average reduction to re-establish 180° commissural angle based on intraoperative assessment; however, the exact width was adjusted according to the measured commissural angle (smaller when near 170° and larger when <150°). In most cases, aortotomy was closed using a vascular prosthesis to shorten the aortic circumference at the level of STJ: Intergard Woven Thoracic Aortic Graft (Getinge, Gothenburg, Sweden; 18 cases), Triplex graft (Terumo, Tokyo, Japan; 11 cases), or J-Graft (Japan Lifeline, Tokyo, Japan; 9 cases). The distal edge of the prosthetic graft was tailored to achieve final 180° commissural alignment.^21^ The intervening Valsalva wall was not additionally plicated, as STJ reduction alone was sufficient to normalize cusp coaptation and leaflet geometry.

### Statistical analysis

Given the retrospective, non-randomized study design, the presence of baseline differences between the groups, and differences in calendar time of patient enrolment, formal hypothesis testing and causal inference were not considered appropriate. Continuous variables are presented as the mean ± SD, and categorical variables are presented as absolute numbers with percentages. Baseline characteristics were compared between the groups using absolute standardized mean differences to illustrate the magnitude of imbalance rather than statistical significance.

To address the influence of confounders in comparison of peak PG and Vmax between the SP and NSP groups, we first calculated their % change between preoperative and 1-year measurements for each patient, calculated between-group differences of their means adjusted for potential confounders, and presented with 95% CIs to reflect the magnitude and uncertainty of the observed associations.

Regression adjustment included clinically relevant covariates selected *a priori*, including age, sex, preoperative aortic root dimensions (AVJ, sinus of Valsalva, STJ diameter), left ventricular function and size (EF, LVEDd, LVEDs), AR grade, baseline peak PG, Vmax, AVA, date of surgery, and hospital treated as a binary variable (Jikei University Hospital vs other institutions) because of the limited number of cases at each participating centre.

All data were analysed using JMP Student Edition version 19.0.1 (SAS Institute, Cary, NC, United States). No formal statistical hypothesis testing was performed.

## RESULTS

The study population consisted of 14 patients with BAV undergoing valve repair with SP (SP group) and 27 patients with BAV treated without SP (NSP group). Concomitant surgery was performed in 1 case each in the NSP group (pulmonary vein isolation) and the SP group (atrial septal defect closure). Baseline patient characteristics are summarized in **Table 1**. Degree of AR was moderate in 3 patients (21.4%) and severe in 11 patients (78.6%) in the SP group, and moderate in 17 patients (63.0%) and severe in 10 patients (37.0%) in the NSP group. BAV phenotype distribution according to Sievers classification^22^ was 2 patients (4.9%) with type 0, 39 patients (95.1%) with type 1, and no patients with type 2. Operative characteristics of the SP and NSP groups are shown in **Table 2**. External suture annuloplasty was performed in all cases. The mean diameter of suture annuloplasty was 22.6 ± 1.0 mm in the SP group and 22.3 ± 1.8 mm in the NSP group. The mean size of vascular prosthesis used to shorten the aortic circumference at the STJ was 23.0 ± 1.4 mm in the SP group and 24.1 ± 1.7 mm in the NSP group. Central plication was performed in 39 of 41 patients, anti-bulging sutures in 27, triangular resection in 5, autologous pericardial patch in 4, and commissural suspension in 4. No patients died in the hospital or during 1-year follow-up in either group. There were no aortic valve reoperations due to AR progression during 1-year follow-up in either group.

**Table 1.** Baseline Patient Characteristics

	Total (*n* = 41)	NSP group (*n* = 27)	SP group (*n* = 14)	ASMD
Age (years)	33.3 ± 11.5	32.3 ± 12.7	36.6 ± 10.3	0.36
Male sex	40 (97.6)	26 (96.3)	14 (100)	0.28
Body surface area (m^2^)	1.82 ± 0.14	1.82 ± 0.18	1.83 ± 0.17	0.05
Medical history				
Hypertension	2 (4.9)	2 (7.4)	0 (0)	0.40
Diabetes mellitus	0 (0)	0 (0)	0 (0)	NA
Chronic obstructive pulmonary disease	0 (0)	0 (0)	0 (0)	NA
Chronic kidney disease	0 (0)	0 (0)	0 (0)	NA
Cerebrovascular disease	0 (0)	0 (0)	0 (0)	NA
Connective tissue disorder	1 (2.4)	1 (3.7)	0 (0)	0.28
Aortic regurgitation				0.93
Moderate	20 (48.8)	17 (63.0)	3 (21.4)	
Severe	21 (51.2)	10 (37.0)	11 (78.6)	
Bicuspid phenotype				1.00
Type 0 (no raphe)	2 (4.9)	2 (7.4)	0 (0)	
Type 1 (1 raphe)	39 (95.1)	25 (92.6)	14 (100)	
R-L fusion	32 (78.0)	18 (66.7)	14 (100)	
R-N fusion	5 (12.2)	5 (18.5)	0 (0)	
L-N fusion	2 (4.9)	2 (7.4)	0 (0)	
Type 2 (2 raphes)	0 (0)	0 (0)	0 (0)	

Abbreviations: ASMD, absolute standardized mean difference; NA, not applicable; SP, sinus plication.

**Table 2.** Intraoperative Characteristics

	Total (*n* = 41)	NSP group (*n* = 27)	SP group (*n* = 14)
Operation			
Aortic cross-clamp time (minutes)	112.7 ± 22.4	119.2 ± 31.5	99.8 ± 7.5
Cardiopulmonary bypass time (minutes)	144.2 ± 20.7	152.3 ± 39.4	128.8 ± 13.2
Suture annuloplasty size (mmutes)	22.4 ± 1.6	22.3 ± 1.8	22.6 ± 1.0
Graft size (mm)	23.7 ± 1.6	24.1 ± 1.7	23.0 ± 1.4
Cusp repair technique			
Central plication	39 (95.1)	25 (92.6)	14 (100)
Anti-bulging suture	27 (65.9)	17 (63.0)	10 (71.4)
Triangle resection	5 (12.2)	3 (11.1)	2 (14.3)
Pericardial patch	4 (9.8)	4 (14.8)	0 (0)
Commissural suspension	4 (9.8)	3 (11.1)	1 (7.1)

Abbreviations: NA, not applicable; SP, sinus plication.

### Preoperative haemodynamic parameters

Preoperative transthoracic echocardiographic parameters are shown in **Table S1**. With regard to the aortic valve, preoperative peak PG was 19.4 ± 6.7 mmHg in the SP group and 22.7 ± 10.4 mmHg in the NSP group. Mean Vmax was 2.21 ± 0.41 and 2.36 ± 0.65 m/s, respectively. Preoperative commissural orientation of non-fused cusp ranged from 120° to 170° with a mean of 144.9° ± 20.6° (144.3° ± 14.5° in the SP group and 145.2° ± 23.7° in the NSP group).

### Postoperative haemodynamic parameters

**Figure 2.**
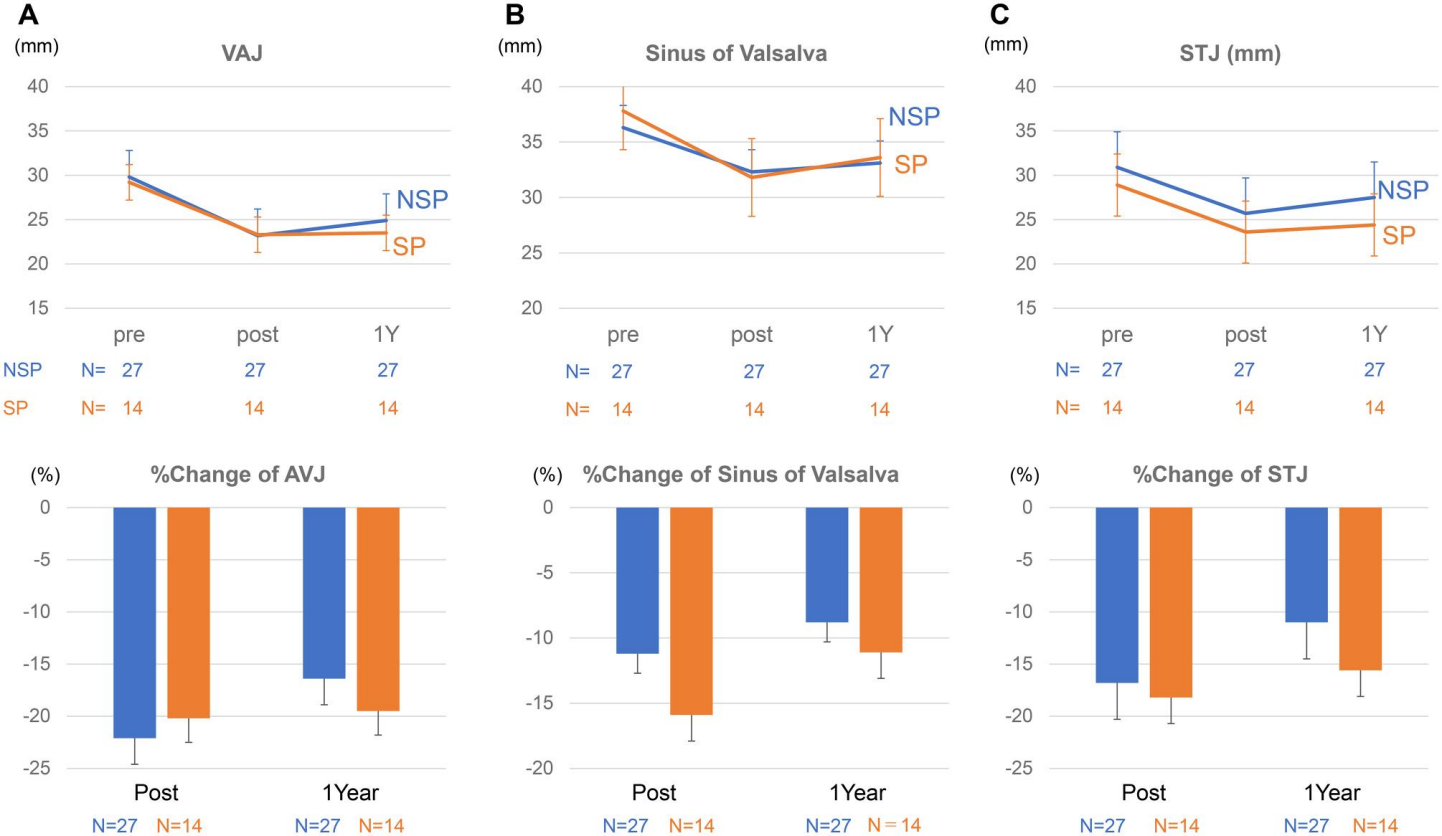
Preoperative and Postoperative (at Discharge and 1 Year) VAJ, the Sinus of Valsalva, and STJ. Diameter (A-C, upper) and percentage change (A-C, lower) of VAJ, the sinus of Valsalva, and STJ in SP and NSP groups. Postoperative percent reduction in sinus of Valsalva diameter was greater at discharge in the SP group. STJ, sinotubular junction; VAJ, ventriculoaortic junction

**Figure 3.**
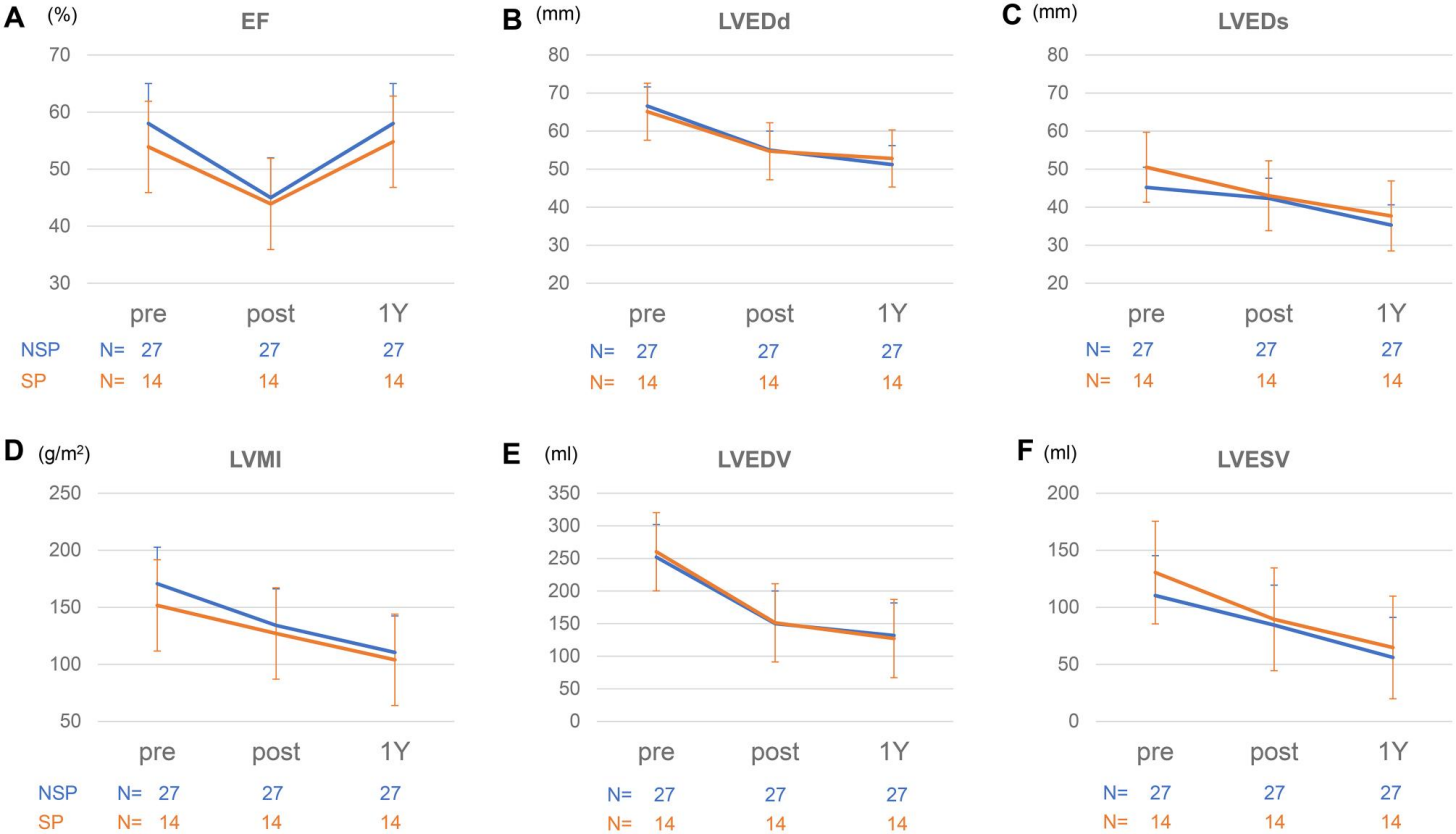
Preoperative and Postoperative Echocardiographic Left Ventricular Parameters. The parameters of left ventricular reverse remodelling, including ejection fraction (EF) (A), left ventricular end-diastolic diameter (LVEDd) (B), left ventricular end-systolic diameter (LVEDs) (C), left ventricular mass index (LVMI) (D), left ventricular end-diastolic volume (LVEDv) (E), and left ventricular end-systolic volume (LVESv) (F) showed no clear differences between groups. LVEDd, LVEDs, LVMI, LVEDv, and LVESv decreased in both the groups after valve repair

Postoperative echocardiographic data at discharge and 1-year follow-up are shown in **Table S2**. Mean diameter of VAJ, sinus of Valsalva, and STJ were comparable between the SP and NSP groups throughout follow-up (**Figure 2A-C**). Postoperative percentage reduction in sinus of Valsalva diameter was greater in the SP group than NSP the group (−15.9% ± 1.9% vs −11.0% ± 1.3% at discharge), whereas no clear between-group differences were observed in % change of VAJ or STJ. Although mean LVEF worsened from 56.6% ± 6.7% preoperatively to 44.6% ± 10.8% at discharge in both the groups, it improved to 56.9% ± 8.6% at 1-year follow-up, equivalent to the level before surgery (**Figure 3A**). LVEDd, LVEDs, and LVMI decreased in both the groups after valve repair (**Figure 3B-D**). LVEDv and LVESv also decreased at discharge and 1-year follow-up in both the groups (**Figure 3E and F**).

**Figure 4.**
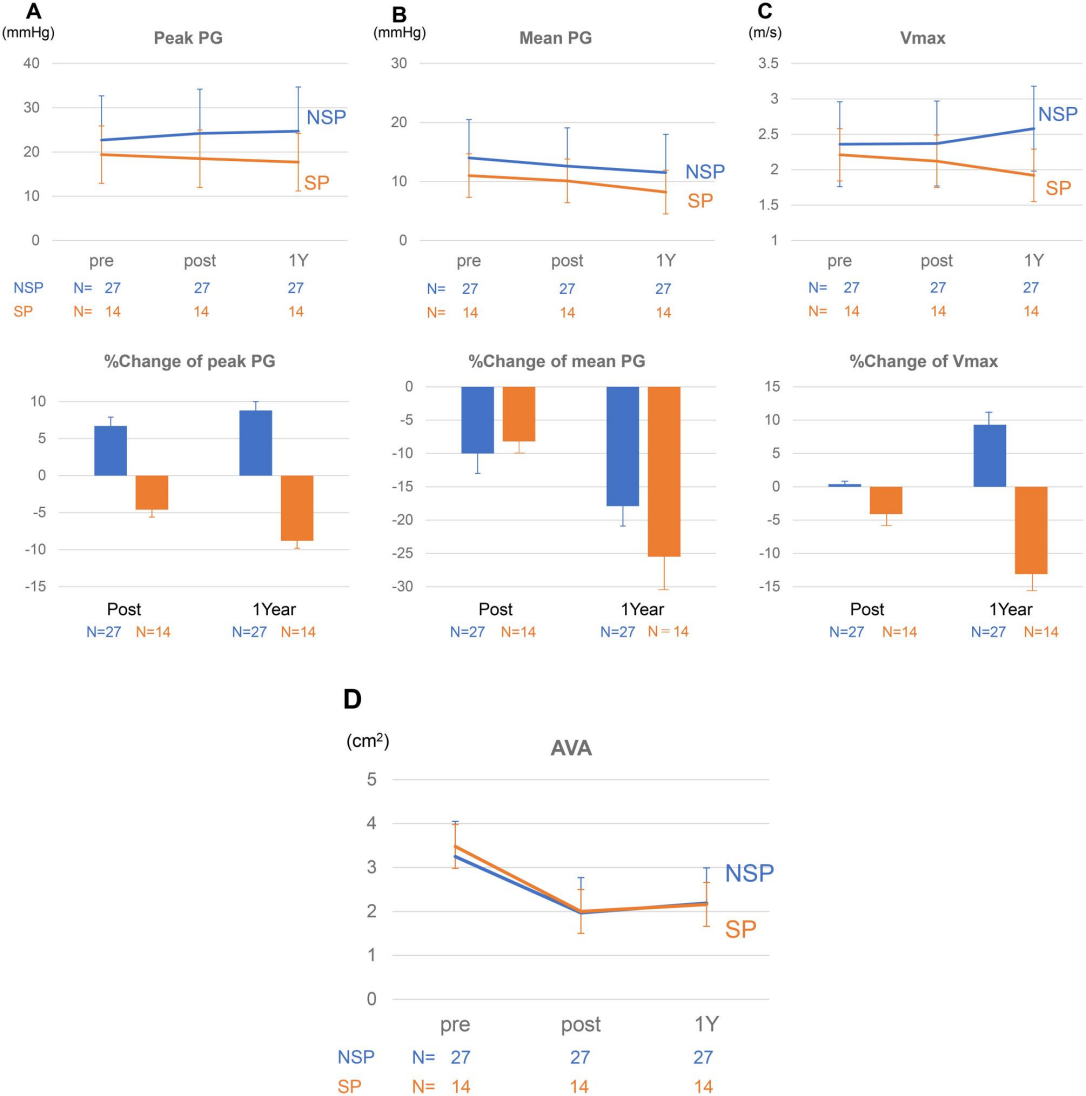
Preoperative and Postoperative Haemodynamic Aortic Valve Parameters. Lower values of peak PG were observed at discharge and at 1 year in the SP group (A). No clear differences were observed in postoperative mean PG or AVA between the groups (B, D). Vmax was lower in the SP group at 1-year follow-up (C). Percentage changes in each parameter are shown in the lower panel. AVA = aortic valve area; NSP = no sinus plication; PG = pressure gradient; SP = sinus plication; Vmax = maximum transvalvular flow velocity

The aortic valves were repaired successfully in all cases with intraoperative mild AR or less in both the groups. In the NSP group, 1 patient showed moderate AR at discharge despite no AR on intraoperative TEE. In the NSP group, unadjusted peak PG increased gradually over time, from 22.7 ± 10.4 mmHg preoperatively to 24.2 ± 9.4 mmHg at discharge and 24.7 ± 9.6 mmHg at 1-year follow-up (**Figure 4A**). The corresponding % changes were +6.6% ± 1.0% at discharge and +8.8% ± 1.3% at 1 year. In contrast, the peak PG decreased over the same period in the SP group, from 19.4 ± 6.4 mmHg preoperatively to 18.5 ± 6.5 mmHg at discharge and 17.7 ± 5.  mmHg at 1 year, with % change −4.6% ± 1.3% at discharge and −8.8% ± 1.1% at 1 year. Mean PG decreased slightly at discharge and 1-year follow-up in both the groups (**Figure 4B**). In the NSP group, Vmax increased from 2.37 ± 0.51 m/s at discharge to 2.58 ± 0.62 m/s at 1 year, whereas it decreased from 2.12 ± 0.37 m/s to 1.92 ± 0.31 m/s in the SP group, respectively (**Figure 4C**). At 1 year, the % change in Vmax relative to baseline was +9.3% ± 2.5% in the NSP group and −13.1% ± 2.1% in the SP group. After converting to % change and applying regression adjustment, no clear between-group differences in peak PG or Vmax were observed at 1-year follow-up. The adjusted mean % difference in peak PG at 1-year follow-up, with the NSP group as the reference, was −11% (95% CI, −63% to +42%) and that for Vmax was −4% (95% CI, −25% to +16%). AVA was 1.97 ± 0.75 cm^2^ at discharge and 2.19 ± 0.81 cm^2^ at 1 year in the NSP group. The respective values in the SP group were 2.00 ± 0.58 and 2.16 ± 0.30 cm^2^. Postoperative AVA values were similar between the groups over follow-up (**Figure 4D**).

## DISCUSSION

Clinical observations showed that commissural orientation <160° and VAJ dilatation are important predictors of repair failure in BAV.^6^ Such asymmetric root geometry results in unbalanced stress distribution on the fused and non-fused cusps, promoting free-margin elongation, not only recurrent AR but also early calcification and development of AS.^23^^,^^24^ As physiological valve opening and closure depend on the integrated geometry of the aortic root, including the sinus of Valsalva, restoration of symmetric root configuration may be critical for durable repair.^8^ In the present study, lower peak PG and Vmax at 1 year were observed in patients who underwent BAV repair with SP. Postoperative transvalvular PG is a key determinant of valve durability, with higher peak PG associated with increased risk of reintervention.^10^^,^^24^ Moreover, PG after BAV repair increases over time and should therefore be minimized immediately after surgery.^25^^,^^26^ Consistent with these observations, patients undergoing BAV repair without SP in our cohort showed gradual increases in peak PG and Vmax, whereas those treated with SP showed lower peak PG and Vmax at 1 year. We speculated that SP corrects asymmetric root geometry, facilitates a more physiological commissural orientation, and reduces cusp tension, thereby improving valve opening and stabilizing valve competence during follow-up. These observations were consistent with the concept that targeted geometric correction, rather than circumferential root reduction, may be sufficient to address the functional asymmetry characteristic of BAV.

The aortic valve should be repaired considering the whole aortic root complex as a 3D structure. Cusp repair alone cannot bring commissural angle closer to 180° following distortion by abnormally high flow stress on cusps.^13^ Adding SP can modify the unbalanced sinus of Valsalva from STJ to the annulus level, resulting in optimal commissural orientation and whole root morphology. Commissural angle showed a moderate negative correlation with peak PG (Spearman’s *ρ* = −0.22, *P *< .001) (**Figure S3**), consistent with the hypothesized mechanism of improved haemodynamics through more symmetrical commissural orientation after SP.^13^ Our preliminary *in vitro* pulsatile flow simulation showed that intercommissural distance is a key factor for valve opening. Shortening of the intercommissural distance seemed to improve mobility of the cusp free margins allowing wider valve opening.^27^ The improved peak PG and Vmax in the SP group despite the absence of clear differences in AVA between the groups may have been due to shortening of intercommissural distance. Further geometric investigations of cusp mobility and root morphology are needed using imaging modalities such as 4D CT. The optimal arrangement of commissural orientation and root morphology from STJ to annulus may play a key role in improving valve stability.

Despite improving peak PG and Vmax, no clear differences were observed in mean PG or AVA during postoperative follow-up between the SP and NSP groups. Although mean PG and AVA did not differ significantly, the observed reductions in Vmax and peak PG after SP may still be clinically relevant. Vmax and peak PG reflect instantaneous maximal transvalvular flow and are sensitive to leaflet opening dynamics and localized cusp stress, whereas mean gradient and calculated AVA integrate the entire Doppler waveform and are influenced by waveform shape and heart rate.^28^ Previous studies showed that elevated early postoperative peak gradients after aortic valve repair are associated with higher reintervention risk. Vohra *et al* reported that patients with postoperative peak AV gradient ≥20 mmHg had significantly higher rates of valve reintervention during follow-up than those with lower gradients.^24^ These data suggest that lowering peak velocity/gradient may reflect more favourable leaflet mechanics and reduced cusp stress, which could translate into improved long-term durability even in the absence of immediate changes in mean gradient or AVA. Differences in mean PG may become more apparent with accumulation of additional data.

This study had several limitations. First, it was a retrospective analysis with a relatively small sample size and short follow-up period. Postoperative echocardiographic and clinical follow-up were analysed up to 1 year after surgery. As SP was introduced from 2021, longer term data were available only for a small subset of patients. Therefore, 1-year follow-up was selected to allow consistent comparison between the groups. Longer follow-up is needed to assess durability of SP and long-term impacts on valve function and reintervention rates. In addition, % changes in peak PG and Vmax were lower in the SP group at 1-year, but their adjusted mean differences were small and associated with wide confidence intervals (peak PG: −11%; 95% CI, −63% to +42%, Vmax: −4%, 95% CI −25% to +16%). Increasing the sample size would allow more precise estimation of between-group differences and may help clarify whether the observed numerical decreases represent a consistent pattern. Further studies with long-term follow-up and larger sample sizes are needed to confirm our findings. Second, SP has been performed extensively in recent cases, with precautions taken to avoid postoperative stenosis. With increasing understanding of BAV anatomy, high-risk cases may now be assigned to replacement. Therefore, factors other than SP, such as surgical era and learning curve, may have contributed to the favourable haemodynamics in the SP group. To confirm our findings, further studies with random use of SP over the same period are required. Third, there was some heterogeneity between the groups. While we used regression adjustment to compare % changes in peak PG and Vmax, matching methods, for example, propensity score matching, would likely allow further reducing bias in estimation. This was not feasible due to the limited cohort size, but will be addressed in future studies with larger case numbers. Fourth, this was a multicentre study. Operations in the NSP group were performed exclusively in the central institution, whereas those in the SP group were performed in both the central institution and 7 associated hospitals. Therefore, interinstitutional differences in operative details or postoperative care may have influenced the outcomes. All procedures were performed by a single surgeon (T.K.) using a consistent technique, thus minimizing potential bias from operator-related differences.

This multicentre observational study suggested that SP may be associated with improved haemodynamic parameters 1 year after asymmetric BAV repair. These findings were hypothesis generating and warrant confirmation in larger prospective studies.

## Supplementary Material

ivag103_Supplementary_Data

## Data Availability

The data are available in the article and online Supplementary Material.

## ABBREVIATIONS

AR: Aortic regurgitation
AS: Aortic stenosis
AVA: Aortic valve area
AVP: Aortic valvuloplasty
BAV: Bicuspid aortic valve
eH: Effective height
gH: Geometric height
LVEDd: Left ventricular end-diastolic diameter
LVEDs: Left ventricular end-systolic diameter
LVEDv: Left ventricular end-diastolic volume
LVESv: Left ventricular end-systolic volume
LVEF: Left ventricular ejection fraction
LVMI: Left ventricular mass index
PG: Pressure gradient
SP: Sinus plication
STJ: Sinotubular junction
VAJ: Ventriculoaortic junction
Vmax: Maximum transvalvular flow velocity
