# Supplementary material for: Haemodynamic Early Outcomes of Sinus Plication for Bicuspid Aortic Valve Repair
Source: Interdiscip Cardiovasc Thorac Surg. 2026 Apr 10;41(4):ivag103. doi: 10.1093/icvts/ivag103 (PMC13125754; doi:10.1093/icvts/ivag103)
Supplement: ivag103_Supplementary_Data [file ivag103_supplementary_data.zip › Supplementary_Data/Supplementary table S1[AU].docx]

**S1.  Preoperative echocardiographic parameters.**

|  | Total  (n=41) | NSP group  (n=27) | SP group  (n=14) | ASMD |
| --- | --- | --- | --- | --- |
| Aortic root |  |  |  |  |
| VAJ (mm) | 29.6 ± 2.9 | 29.8 ± 2.9 | 29.2 ± 3.1 | .16 |
| Sinus of Valsalva (mm) | 37.5 ± 8.4 | 36.3 ± 2.5 | 37.8 ± 9.3 | .38 |
| STJ (mm) | 30.5 ± 4.5 | 30.9 ± 4.9 | 28.9 ± 2.5 | .55 |
| Left ventricle |  |  |  |  |
| LVEDd (mm) | 66.1 ± 8.1 | 66.6 ± 6.1 | 65.1 ± 11.1 | .16 |
| LVEDs (mm) | 47.0 ± 7.8 | 45.2 ± 5.3 | 50.5 ± 10.6 | .63 |
| LVEDv (ml) | 253.7 ± 81.6 | 252.0 ± 84.0 | 260.3 ± 78.1 | .10 |
| LVESv (ml) | 114.6 ± 46.4 | 110.3 ± 45.7 | 130.5 ± 49.1 | .43 |
| LVMI (g/m^2^) | 166.8 ± 43.2 | 170.7 ± 45.7 | 151.7 ± 29.5 | .49 |
| LVEF (%) | 56.6 ± 6.7 | 58.0 ± 6.4 | 53.9 ± 6.9 | .60 |
| Aortic valve |  |  |  |  |
| peak PG (mmHg) | 21.6 ± 8.8 | 22.7 ± 10.4 | 19.4 ± 6.7 | .36 |
| mean PG (mmHg) | 13.0 ± 7.1 | 14.0 ± 8.2 | 11.0 ± 4.1 | .46 |
| Vmax (m/s) | 2.30 ± 0.58 | 2.36 ± 0.65 | 2.21 ± 0.41 | .27 |
| AVA (cm^2^) | 3.33 ± 1.15 | 3.25 ± 1.20 | 3.48 ± 1.06 | .20 |
| Commissure angle (degree) | 144.9± 20.6 | 145.2 ± 23.7 | 144.3 ± 14.5 | .79 |

ASMD: absolute standardized mean difference, AVA: aortic valve area, VAJ: ventriculoaortic junction, LVEDd: left ventricular end-diastolic diameter, LVEDs: left ventricular end-systolic diameter, LVEDv: left ventricular end-diastolic volume, LVESv: left ventricular end-systolic volume, LVEF: left ventricular ejection fraction, LVMI: left ventricular mass index, PG: pressure gradient, STJ: sinotubular junction, Vmax: maximum transvalvular flow velocity.
