# Supplementary material for: Haemodynamic Early Outcomes of Sinus Plication for Bicuspid Aortic Valve Repair
Source: Interdiscip Cardiovasc Thorac Surg. 2026 Apr 10;41(4):ivag103. doi: 10.1093/icvts/ivag103 (PMC13125754; doi:10.1093/icvts/ivag103)
Supplement: ivag103_Supplementary_Data [file ivag103_supplementary_data.zip › Supplementary_Data/Supplementary table S2[AU].docx]

**S2.  Unadjusted postoperative echocardiographic parameters.**

|  | Total  (n=41) | NSP group (n=27) | SP group  (n=14) | *p* value |
| --- | --- | --- | --- | --- |
| VAJ (mm) -pre | 29.6 ± 2.9 | 29.8 ± 2.9 | 29.2 ± 3.1 |  |
| -post | 23.2 ± 2 .5 | 23.2 ± 2.6 | 23.3 ±2.0 | .863 |
| -1 year | 24.6 ± 2.8 | 24.9 ± 3.0 | 23.5 ± 1.4 | .064 |
| Valsalva (mm)-pre | 37.5 ± 8.4 | 36.3 ± 2.5 | 37.8 ± 9.3 |  |
| -Post | 31.9 ± 3.3 | 32.3 ± 1.7 | 31.8 ± 3.6 | .642 |
| -1 year | 33.5 ± 2.8 | 33.1 ± 1.8 | 33.6 ± 3.0 | .559 |
| STJ (mm) -pre | 30.5 ± 4.5 | 30.9 ± 4.9 | 28.9 ± 2.5 |  |
| -Post | 25.3 ± 2.9 | 25.7 ± 2.7 | 23.6 ± 3.4 | .176 |
| -1 year | 26.9 ± 4.1 | 27.5 ± 4.1 | 24.4 ± 3.5 | .089 |
| LVEDd (mm) -pre | 66.1 ± 8.1 | 66.6 ± 6.1 | 65.1 ± 11.1 |  |
| -Post | 54.9 ± 8.6 | 55.0 ± 5.2 | 54.7 ± 7.5 | .902 |
| -1 year | 51.7 ± 4.7 | 51.2 ± 2.9 | 52.8 ± 7.2 | .437 |
| LVEDs (mm)-pre | 47.0 ± 7.8 | 45.2 ± 5.3 | 50.5 ± 10.6 |  |
| -Post | 42.5 ± 7.7 | 42.3 ± 7.1 | 43.0 ± 9.1 | .787 |
| -1 year | 35.9 ± 5.2 | 35.3 ± 3.5 | 37.7 ± 8.3 | .392 |
| LVEDv (ml)-pre | 253.7 ± 81.6 | 252.0 ± 84.0 | 260.3 ± 78.1 |  |
| -post | 150.3 ± 47.5 | 150.0 ± 45.1 | 151.2 ±59.9 | .962 |
| -1 year | 130.7 ± 36.3 | 131.8 ± 30.1 | 127.1 ± 54.7 | .821 |
| LVESv (ml) -pre | 114.6 ± 46.4 | 110.3 ± 45.7 | 130.5 ± 49.1 |  |
| -post | 85.5 ± 41.8 | 84.5 ± 36.4 | 89.5 ± 61.9 | .843 |
| -1 year | 58.1 ± 23.4 | 56.1 ± 16.5 | 64.8 ± 39.8 | .563 |
| LVMI (g/m^2^) -pre | 166.8 ± 43.2 | 170.7 ± 45.7 | 151.7 ± 29.5 |  |
| -post | 132.8 ± 36.1 | 134.2 ± 32.6 | 127.1 ± 50.2 | .730 |
| -1 year | 109.0 ± 33.8 | 110.5 ± 32.7 | 104.0 ± 39.5 | .682 |
| LVEF (%) -pre | 56.6 ± 6.7 | 58.0 ± 6.4 | 53.9 ± 6.9 |  |
| -post | 44.6 ± 10.8 | 45.0 ± 11.1 | 43.9 ± 10.5 | .758 |
| -1 year | 56.9 ± 8.6 | 58.0 ± 6.9 | 54.8 ± 11.3 | .349 |
| Aortic valve |  |  |  |  |
| AR grade -post | |  |  | .717 |
| None | 23 (56.1%) | 15 (55.6%) | 9 (64.3%) |  |
| Trace | 16 (39.0%) | 11 (40.7%) | 5 (35.7%) |  |
| Mild | 0 (0%) | 0 (0%) | 0 (0%) |  |
| Moderate | 1 (2.5%) | 1 (3.8%) | 0 (0%) |  |
| Severe | 0 (0%) | 0 (0%) | 0 (0%) |  |
| AR grade -1 year | |  |  | .489 |
| None | 16 (40.0%) | 9 (34.6%) | 7 (50.0%) |  |
| Trace | 15 (35.0%) | 10 (37.0%) | 6 (42.9%) |  |
| Mild | 8 (20.0%) | 7 (26.9%) | 1 (7.1%) |  |
| Moderate | 1 (2.5%) | 1 (3.8%) | 0 (0%) |  |
| Severe | 0 (0%) | 0 (0%) | 0 (0%) |  |
| Peak PG (mmHg)-pre | 21.6 ± 8.8 | 22.7 ± 10.4 | 19.4 ± 6.7 |  |
| -post | 22.2 ± 8.9 | 24.2 ± 9.4 | 18.5 ± 6.5 | .032 |
| -1 year | 22.6 ± 9.7 | 24.7 ± 9.6 | 17.7 ± 5.5 | .040 |
| Mean PG (mmHg)-pre | 13.0 ± 7.1 | 14.0 ± 8.2 | 11.0 ± 4.1 |  |
| -post | 11.8 ± 6.1 | 12.6 ± 6.9 | 10.1 ± 3.5 | .141 |
| -1 year | 10.0 ± 5.3 | 11.5 ± 5.9 | 8.2 ± 3.5 | .297 |
| Vmax (m/s) -pre | 2.30 ± 0.58 | 2.36 ± 0.65 | 2.21 ± 0.41 |  |
| -post | 2.28 ± 0.48 | 2.37 ± 0.51 | 2.12 ± 0.37 | .088 |
| -1 year | 2.38 ± 0.62 | 2.58 ± 0.62 | 1.92 ± 0.31 | <.001 |
| AVA (cm^2^) -pre | 3.33 ± 1.15 | 3.25 ± 1.20 | 3.48 ± 1.06 |  |
| -post | 1.98 ± 0.70 | 1.97 ± 0.75 | 2.00 ± 0.58 | .454 |
| -1 year | 2.18 ± 0.71 | 2.19 ± 0.81 | 2.16 ± 0.30 | .231 |

AR: aortic regurgitation, AVA: aortic valve area, VAJ: ventriculoaortic junction, LVEDd: left ventricular end-diastolic diameter, LVEDs: left ventricular end-systolic diameter, LVEDv: left ventricular end-diastolic volume, LVESv: left ventricular end-systolic volume, LVEF: left ventricular ejection fraction, LVMI: left ventricular mass index, PG: pressure gradient, STJ, sinotubular junction, Vmax: maximum transvalvular flow velocity. *P* values for categorical variables were obtained using Chi-square tests, as appropriate.
