# Supplementary figures and images for: Haemodynamic Early Outcomes of Sinus Plication for Bicuspid Aortic Valve Repair
Source: Interdiscip Cardiovasc Thorac Surg. 2026 Apr 10;41(4):ivag103. doi: 10.1093/icvts/ivag103 (PMC13125754; doi:10.1093/icvts/ivag103)

## Slide 1
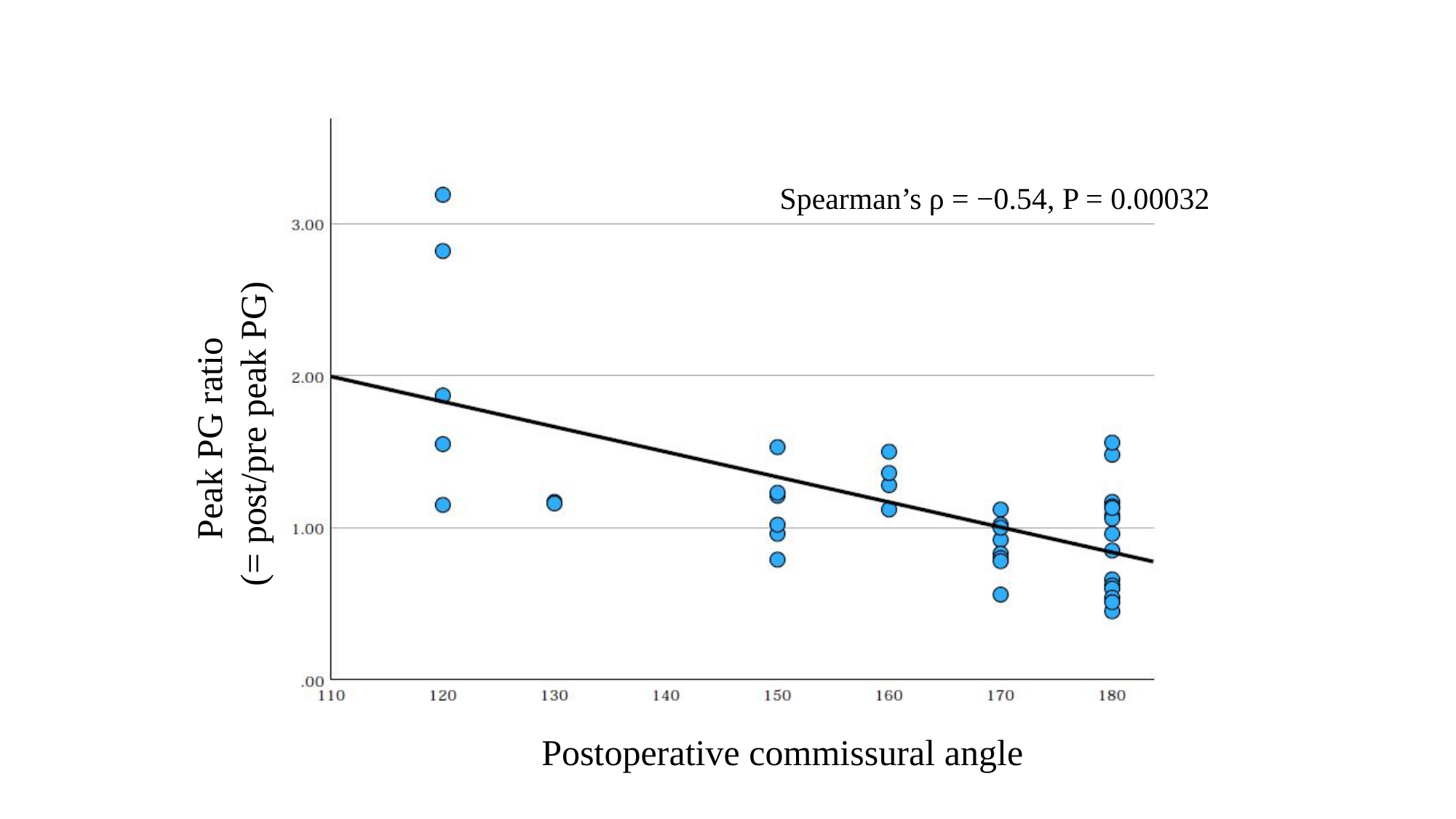

Spearman’s ρ = −0.54, P = 0.00032
Peak PG ratio
 (= post/pre peak PG)
Postoperative commissural angle

Supplement: ivag103_Supplementary_Data [file ivag103_supplementary_data.zip › Supplementary_Data/Supplementary figure S3.pptx]
